# Correction: Programme for the Effective Promotion of Maternal Psychosocial Wellbeing (PREPWELL) in Ghana: Development and field-testing of a mHealth Intervention in a rural setting

**DOI:** 10.1371/journal.pmen.0000642

**Published:** 2026-06-12

**Authors:** Benedict Weobong, Solomon Nyame, Dzifa Attah, Kenneth Ae-Ngibise, Joseph Osafo, Betty Kirkwood, Angela Ofori-Atta, Kwaku Poku Asante, Philip Baba Adongo

The corresponding author’s email address is incorrect. Benedict Weobon’s email address is: bweobong@yorku.ca.
