# Supplementary material for: Programme for the Effective Promotion of Maternal Psychosocial Wellbeing (PREPWELL) in Ghana: Development and field-testing of a mHealth Intervention in a rural setting
Source: PLOS Ment Health. 2026 May 19;3(5):e0000571. doi: 10.1371/journal.pmen.0000571 (PMC13186332; doi:10.1371/journal.pmen.0000571)
Supplement: S1 Text — (PDF) [file pmen.0000571.s001.pdf]

PRogramme for the Effective Promotion of maternal psychosocial WELL being (PREPWELL)- intervention development

Sequence of research activities:

The planned sequence of the research activities will be:

1. Further pretesting of all the informed consent forms and the other study instruments with a small number of suitable people to ensure that they are well understood in the language that they will be administered in, with modification where needed. This will be particularly important for the mothers and husbands.
2. Selection of the 2 communities for the study.
3. Identifying/stakeholder mapping to identify Key Informants (KI) (community health nurses, CMHO, CPNs, psychologists, health directors/administrators, husbands/partners) and mothers.
4. Making arrangements to invite KIs for interviews and obtaining written informed consent prior to the scheduled interviews.
5. Making arrangements to invite pregnant or mothers with children up to 1 year to the series of participatory workshops and obtaining initial agreement.
6. First set of participatory workshops with pregnant or mothers with children up 1 year.
7. Conduct the key informant interviews, after obtaining the respondent’s written informed consent.
8. **The second** set of participatory workshops with pregnant or mothers with children up 1 year.
9. Further key informant interviews or specific questions to the informants, if required.
10. Consensus and ToC Participatory workshop with the key informants (community health nurses, CMHO, CPNs, psychologists, health directors/administrators, husbands/partners).
11. Finalisation of the basic architecture of PREPWELL.

PREPWELL Study Instrument:

Semi-structured interviews with Key Informants

Objectives of the Interview

(Note: The relative importance of each of these objectives will differ between key informants, based on their organization and specific expertise)

1. Obtain factual information on what relevant mental health and psychosocial services are already in place for mothers.
2. Obtain the opinions of key informants on the feasibility, acceptability, and range of mental health and psychosocial wellbeing services that they think should be provided during routine antenatal/postnatal visits for pregnant women and women who have recently delivered.
3. Collect the opinions of key informants on how best such routine mental health and psychosocial wellbeing services might be delivered using technology.

Interview process: Interviews should be tailored to the respondents' area of expertise. The key informant should have been sent the questions below in advance so that they could have the chance to prepare for the interview. The interviewer should obtain informed consent using ICF ***00.07*** which includes asking their permission to audio record the interview. If a Key Informant wants to invite one or more of their colleagues to join the interview, that is fine, but each person completes an informed consent form.

The interview should begin with open-ended questions to allow the respondent to provide their perspective without prompting (suggesting potential answers to) them. As the interview progresses, the questions can become more specific to ensure that full use is made of the key informant's relevant expertise.

Guide and script for interviews with key informants

Section 1: Introduction & Informed Consent

See ICF ***00.07***

If permission has been granted for audio recording, switch on the recorder. State your name and the date and use a coding system to identify the person being interviewed. Whether or not audio recording is permitted, the interviewing team should write down the key points during the interview. The name and title of the key informant should not be included in reports for privacy reasons.

Section 2: Interview questions, to be adapted to key informant context

1. What health care services are provided to pregnant and mothers who have recently delivered in the Kintampo Area?
    a. *Can you provide examples of the type of services provided?*
    b. *Please also elaborate on where these services are most often offered (such as in health facilities, through outreach services, etc.).*
    c. *What organizations provide each service?*
2. Would you say that these services are offered equally across groups of mothers (such as mothers from high socioeconomic status (SES) families vs. low SES, or older vs. younger mothers)?
    a. *If there are discrepancies, can you elaborate on why they exist?*

    Do you think mothers or pregnant women have mental health problems?
3. If they have not mentioned mental health/psychosocial health service, ask: "Is there a mental health/psychosocial health service programme in the Kintampo Area?
    a. *If yes, explore further eg. What is included? What organization(s) are involved and for which aspects?*
    b. *Are there special services for mothers?*
    c. *Who is providing the services?*
    d. *To whom?*
    e. *How are they delivered?*
    f. *What do they include?*

4. What systems are there for referring mothers with a mental health/psychosocial challenge (e.g. depression, anxiety) from community/primary care to a community specialist?
    a. *Please provide examples if possible.*

b. How functional are they?

5. Do mothers with a mental health/psychosocial challenge (e.g. depression, anxiety) have to pay out-of-pocket for all the health services that they receive?
    a. *If not, which ones are free? And which are covered by health insurance?*
6. Are you aware of any programme that is providing routine mental health/psychosocial services (e.g. depression, anxiety) for mothers in the Kintampo Area? *If so, please give details such as:*
7. We are planning to develop a programme to support mothers to acquire essential skills in order to take care of **their own mental health** during pregnancy. What do you think about this idea?
    a. *Explore.*
8. If such a routine health programme were to be introduced, what specific things or areas should be included to help mothers take care of their own mental health?
    a. *Why? Explore.*
9. In this programme, we plan to provide these essential skills to mothers through their mobile phones.
    a. *What do you think about this idea?*
    b. *Do you think it is feasible? Why?*
    c. *Do you think it would be acceptable? Why?*
    d. *How do you think we could make this possible?*
10. Assuming this programme to support mothers is available, accessible and acceptable to mothers, are there any reasons why mothers may still not use this service?
    a. *Please explain and give examples.*
11. Finally, do you have any further suggestions for the **content** or **delivery** of the planned routine health programme to support mothers to acquire essential skills in order to take care of **their own mental health** during pregnancy and after delivery?

PREPWELL Study Instrument:

Series of Participatory Workshops for pregnant women and mothers with children up to 1-year-old

1. Objectives of the Workshops

1. To explore with pregnant women and mothers with children up to **1-year-old** living in the two selected communities for PREPWELL whether they think the proposal to introduce a routine health programme to support mothers to acquire essential skills in order to take care of **their own mental health** during pregnancy and after delivery is a good idea.
2. To check with pregnant women and mothers with children up to **1-year-old** whether the team's developing ideas and plans for the design of the PREPWELL intervention would be attractive and perceived as being useful to pregnant women and mothers with children up to **1-year-old** like themselves.

2. Background

The series of two workshops will be iterative and should be separated by at least one week (usually longer than this). They will use the principles of person-centred design, in which ideas that are generated in earlier sessions are worked on by the investigators and presented back to the group for further discussion and refinement at the next workshop. The workshops will use participatory learning and action (PLA) techniques in order to actively engage the participants and make the sessions fun.

3. Action Required Before the First Workshop

3.1. Selection of mothers for participation in the workshops

In each of the two communities selected for the study, twenty mothers (10 pregnant; 10 recently delivered) will be invited to participate in a series of two participatory workshops to discuss the potential design for the PREPWELL intervention. These mothers can be sampled from the HDSS database, oversampling for possible refusals.

3.2. Preparations for the first workshop

The team will need to identify a suitable venue (see below) in which to hold the workshops. This should be as close as possible to where the mothers live.

4. Facilitators, Room, Equipment and Supplies

4.1.Facilitators: At least two facilitators are needed for each workshop. Ideally**.** the lead facilitator should have used (or at least participated in a session using) visualization in participatory programmes (VIPP) previously. All facilitators should have read the instructions and hints related to the use of VIPP given in Annex 1 to this document. At least one of the facilitators should be taking notes at all times during the workshop. Remember that the comments made should not be attributable to a specific participant in these notes.

4.2.**Room:** The workshops should be conducted in a room that can comfortably hold up to 15 people and with enough space and flexibility to allow group work using VIPP and other active PLA techniques in addition to presentations using flip charts.

4.3.Equipment and supplies:

- PowerPoint projector and screen
- Movable seats for up to 15 people
- Two flip chart stands and flip charts
- 10 dark blue or black marker pens – one per participants plus a few spares (fewer if the expected number of participants is less than this)
- 2 red marker pens (for title cards)
- 100 VIPP cards, 50 of each of two different pale colours. VIPP cards are pale coloured cards that are **one-third** of an A4 sheet (ie 210mm (portrait width) by approx. 100mm).
- One large, highly visible "sticky" display board or a suitable area of wall or window that the VIPP cards can be stuck on to using Blu tack or something similar (eg. *UHU patafix deco*).
- At least one sheet of paper and a ballpoint pen for each participant.
- 120 small round(ish) stones – not so round that they can roll.
- Two boxes with a small hole in the top, just large enough to allow a stone to enter the box. One box should be marked "YES" and the other should be marked "No".
- A screen
- On the flip chart stand, there should be sheets with the following information on it:

- **Common mental health disorders, including severe mental health disorders, and alcohol use:**

1. Depression
2. Anxiety
3. Stress
4. Psychosis
5. Alcohol misuse

- **Six psychosocial well-being areas:**

1. Autonomy
2. Environmental mastery
3. Personal growth
4. Relations with others
5. Purpose in life
6. Self-acceptance

- Core Healthy Activity Programme HAP) areas:

1. Understanding the HAP and how to apply it to daily life
2. Learning together (identifying causes and solutions to poor mental health/low mood/poor psychosocial well-being) during pregnancy/after delivery
3. Goal-setting
4. Involving a Significant Other
5. Doing homework/action plan
6. Problem-solving/dealing with barriers
7. Doing pleasurable activities
8. Dealing with rumination

These sheets should be covered at the start of the session.

- Non-alcoholic drinks – enough for one for each participant
- Snacks for the participants.

3. **Workshop process**

All the participants should have already given informed consent before the workshop. Prior to each workshop, the facilitators should check that there is a signed informed consent form for each of the expected participants.

The two boxes should be placed behind a screen such that the participants cannot see them from where they are sitting.

The facilitators should decide in advance when they propose would be the best time for the participants to receive their drink and snack. This might be as they arrive, partway through the workshop, or at the end.

The workshop will not be audio recorded.

4. Workshop Guide

6.1. First workshop

Section 1: Introductions (15 minutes)

As participants arrive at each workshop, the facilitators should ensure that they register so that a complete record is kept of all participants in the workshop – including those who arrive late. Remember that the study must have signed informed consent from every participant.

The facilitators should introduce themselves and state the objectives of the research as a whole and of this specific workshop. They should mention that it will be participatory and will last up to three hours. They should remind the participants that everything that anyone says should be kept confidential (secret).

The lead facilitator should then ask the participants to speak to one of the other participants that they have not met before for 3 minutes so that they can introduce them to the other participants. During this time, they need to learn their name, organization, job, and one surprising fact about them. The lead facilitator can give an example of a surprising fact about themselves to illustrate this, eg. that they once won a hip hop dance competition at school; that they hate the colour green; etc.

The lead facilitator should then ask each person to introduce the person they were with. Make sure this is done as quickly and efficiently as possible.

Section 2: Interactive Activities (120-150 minutes)

One of the facilitators should present the main objectives of the study, focusing on the fact that some mothers can have important mental health problems that they don't realise they have or that they don't think are important enough for them to go to see a health worker about or that they don't realise that a health worker can help them with. Add that it is also common that during pregnancy or soon after birth, some mothers experience mental health/mood problems that can become important health issues for them and the baby. We are proposing to introduce a routine health programme to support mothers to acquire essential skills in order to take care of **their own mental health** during pregnancy and after delivery. But before doing that, we want to check with them whether they think this would be a good idea and, if so, what they would be keen to see included in this programme and how they would like to have it organized.

During the presentation, the facilitator should make it clear that many different people will be consulted, including their husbands and health care workers.

Activity 1. The first task is for each participant to tell us whether they have gone to see a health worker within the past 3 years on how to take care of **their own mental health** during pregnancy and after delivery or for a mental health problem in general.

First, the facilitator should explain what is meant by "take care of **their own mental health**". This is when someone sees a health worker to discuss something that is bothering her and to get help in terms of advice on how to deal with the problem. This is usually done mostly through talking or what we refer to as counselling.

The facilitator should mention that such information is private, so we plan to do this in a way where nobody will know who said what. To achieve this, each participant will be asked to come to the front of the room to select one stone from the heap and go behind the screen.

They should then place their stone either into the box that says "Yes" on it if they have been to see a health worker within the past 3 years on how to take care of **their own mental health** during pregnancy and after delivery or for a mental health problem in general or into the box that says "No" on it if they haven't been to see a health worker for this purpose.

Everyone should put a stone into one box.

When all the participants have put their stones into a box, the facilitator should bring the boxes to the front and ask two of the participants to open them, to see how many stones there are in each of the two boxes. The numbers should be announced and recorded on a flip chart.

Discuss.

Activity 2. Next, get the participants to repeat the same task, but this time the question is "Would you like the opportunity to receive help on how to take care of **your own mental health** during pregnancy and after delivery or for a mental health problem in general?"

When all the participants have put their stones into a box, the facilitator should bring the boxes to the front and ask two of the participants to open them, to see how many stones there are in each of the two boxes.

Discuss the results and ask participants to expand on what they thought. Why?

Activity 3. What mental health areas would you like this programme to focus on?

Reveal the flip chart paper with the summary of the mental health conditions listed on it. The facilitator should explain in plain language what each of the mental health conditions means.

Place the flip chart paper on the floor.

Each participant should be given 3 stones and they should place one stone on each of the three mental health areas that they would most like to be included in the programme.

Summarize and Discuss. Was anything important missing from the list?

Activity 4. What psychosocial well-being areas would you like this programme to focus on?

Reveal the flip chart paper with the summary of the psychosocial well-being areas listed on it. The facilitator should explain in plain language what each of the psychosocial well-being areas mean**s**.

Place the flip chart paper on the floor.

Each participant should be given 5 stones and they should place one stone on each of the six psychosocial well-being areas that they would most **likely want** to see the programme address.

Summarize and Discuss. Was anything important missing from the list?

Activity 5. What elements of the Healthy Activity Programme would you like this programme to focus on?

Reveal the flip chart paper with the summary of the elements of the Healthy Activity Programme listed on it. The facilitator should explain in plain language what each of the elements of the Healthy Activity Programme mean**s**.

Place the flip chart paper on the floor.

Each participant should be given 5 stones and they should place one stone on each of the eight elements of the Healthy Activity Programme that they would most **likely want** to see the programme focus on.

Summarize and Discuss. Was anything important missing from the list?

Activity 6. What would make PREPWELL i.e the programme on how to take care of **their own mental health** during pregnancy and after delivery or for a mental health problem in general more attractive or useful?

Ask the participants to get into two groups of five. Explain the task to them.

Start by asking anyone to suggest something that would make the PREPWELL programme more attractive to them. Something that would make them want to use the PREPWELL programme on their phones to take care of **their own mental health** during pregnancy and after delivery. Explain that this could be something about how easy to use the PREPWELL programme facility on the phone, what should be included in the PREPWELL programme, the language used, costs issues, or anything else that would make them want to use the PREPWELL programme on their phones. Write the first two suggestions up on a flip chart, but do not allow any discussion or criticism of these.

Tell the groups that they have ten minutes working in their group to come up with up to five suggestions of what would make the PREPWELL programme more attractive to them. If they have more than five suggestions, the group should discuss all of the suggestions and agree on what are the most important five. Someone in the group should write these five down on a piece of paper and be ready to read them out when asked to do so.

At the end of the ten minutes, get each group to read out their suggestions. One of the facilitators should write each suggestion on to a VIPP card and pass it to another facilitator, who sticks it up on the wall, grouping them as they go along. The groups should include: Easy/friendly Technology; Mental Health Areas Covered; Psychosocial Well-being Areas Covered; Costs – but other groupings can also be created.

Summarize and discuss.

Section 3. Wrap-up (15 minutes)

The lead facilitator should then summarize the main outcomes from the workshop, based on each activity.

They should remind the participants that they may be asked to come back for another workshop if necessary.

Finally, thank the participants, the other facilitator(s) and anyone else who helped organize the workshop, and wish the participants a safe journey home.

PREPWELL Study Instrument: Participatory workshop for key informants

1. **Objectives of the Workshop**
    1. To present the results of the study and obtain feedback from the key informants (nurses, CMHO, CPNs, psychologists, health directors/administrators, husbands/partners) and their suggestions on whether the idea of PREPWELL i.e. introducing a routine health programme to support mothers to acquire essential skills in order to take care of **their own mental health** during pregnancy and after delivery is a good idea worth taking forward?
    2. If so:
        a. Is the concept of guided self-help culturally appropriate?
        b. What should be the content of PREPWELL?
        c. Who should be involved in the programme?

2. **Facilitators, Room, Equipment and Supplies**

2.1.Facilitators: At least three facilitators are needed. Ideally**,** the lead facilitator should have used (or at least participated in a session using) visualization in participatory programmes (VIPP) previously. All facilitators should have read the instructions and hints related to the use of VIPP given in Annex 1 to this document. At least one of the facilitators should be taking notes at all times during the workshop. Remember that the comments made should not be attributable to a specific participant in these notes.

2.2.**Room:** The workshop should be conducted in a room that can hold up to 50 people and with enough space and flexibility to allow group work using VIPP in addition to presentations using PowerPoint and flip charts.

2.3.Equipment and supplies:

- PowerPoint projector and screen
- Movable seats for up to 20 people
- Three flip chart stands and flip charts
- 50 dark blue or black marker pens – one per participants plus a few spares (fewer if the expected number of participants is less than this)
- 5 red marker pens (for title cards)

- 100 VIPP cards. VIPP cards are pale coloured cards that are **one-third** of an A4 sheet (ie 210mm (portrait width) by approx. 100mm).
- Three large, highly visible "sticky" display boards or three suitable areas of wall or window that the cards can be stuck on to using Blu tack or something similar (eg. *UHU patafix deco*).
- At least one sheet of paper and a ballpoint pen for each participant.
- Non-alcoholic drinks – enough for one for each participant
- Snacks for the participants.

5. **Workshop process**

The study team should have prepared a brief draft summary report of the study findings that can be given to the participants at the workshop. The report should focus on:

- Evidence of technology-delivered health interventions for mothers.
- Current psychosocial/mental health services available in the Kintampo area.
- Community health services structure in the Kintampo area.
- Feasibility, acceptability, of a mobile phone-delivered routine health programme to support mothers to acquire essential skills in order to take care of **their own mental health** during pregnancy and after delivery.
- **The proposed** content of PREPWELL (mental health conditions to focus on, psychosocial and well-being areas to include, and HAP elements to maintain).

All the key informants should have already given informed consent before they were interviewed. Prior to the workshop, the facilitators should check that there is a signed informed consent form for each of the expected participants where they have agreed to take part in the workshop. If any additional participants attend, informed consent must be obtained from them using ICF ***00.07.***

The facilitators should decide in advance when they propose would be the best time for the participants to receive their drink and snack. This might be as they arrive, **partway** through the workshop, or at the end.

The workshop will not be audio recorded.

6. **Workshop Guide**

Section 1: Introductions and ice-breaker exercise (10 minutes)
As participants arrive, the workshop facilitators should ensure that they register so that a complete record is kept of all participants in the workshop – including those who arrive late. Remember that the study must have written informed consent from every participant, even if they are representing someone else.

At registration, each participant should be given the brief summary report.

The facilitators should introduce themselves and state the objectives of the research as a whole and of this specific workshop. They should mention that it will be participatory and will last about 90 minutes. They should remind the participants that everything that anyone says should be kept confidential (secret).

The lead facilitator should then ask the participants to speak to one of the other participants that they have not met before for 3 minutes so that they can introduce them to the other participants. During this **time,** they need to learn their name, organization, job, and one surprising fact about them. The lead facilitator can give an example of a surprising fact about themselves to illustrate this, eg. that they once won a hip hop dance competition at school; that they hate the colour green; etc.

The lead facilitator should then ask each person to introduce the person they were with. Make sure this is done as quickly and efficiently as possible.

Section 2: Presentation of the study's main findings (20 minutes)
One of the facilitators should then present the main findings of the study, focusing on the results related to the questions that will be the subject of this workshop (see the objectives listed above), and especially the research team's proposals for the content, delivery strategy, and delivery team for routine health programme to support mothers to acquire essential skills in order to take care of **their own mental health** during pregnancy and after delivery. This presentation should not take more than 15 minutes, followed by up to 5 minutes of questions for clarification (NOT discussion comments – these will be captured during the subsequent group exercises).

Section 3: Group work on the implications of the study's findings (30 minutes)
The participants should be divided into three groups of roughly equal size. Each should contain a mixture of community health nurses, community mental health officers, community psychiatric nurses, clinical psychologists, husbands/partners, health directorate officials, assisted by one of the facilitators, who will act as the person who writes the results of the discussion on VIPP cards. Each group should have a pre-determined chair. The chair can either act as the rapporteur themselves or either designate someone else to be the rapporteur or ask for a volunteer for this role. Each group's facilitator should have VIPP cards of a different colour.

The lead facilitator should move between the groups ensuring that they know what they should be doing and are staying on track, both in terms of the task and related to the time available. They should announce when there is five minutes left.

The tasks:
Each group should complete three tasks:

1. Discussion (5 minutes): Should the idea of introducing a routine health programme to support mothers to acquire essential skills in order to take care of **their own mental health** during pregnancy and after delivery be taken forward?
   - The facilitator should write the answer to this question on a VIPP card (following the rules given in Annex 1). If the group wishes to give further details, the facilitator should write these on additional VIPP cards, but still following the rules given in Annex 1. The group should hand these **to** the facilitators who should group them on the wall ready for discussion (see below).
2. Discrete choices (5 minutes): Prior to the workshop, the research team should have prepared up to five discrete choices printed on a sheet of paper. These should relate to the most important choices that the research team need to make. An example could be: "The recruitment of mothers should be done at the ANC or CWC or CHPS compound; who should provide the Guided Self-Help; CHN, CMHO; what should be the frequency of GSH; weekly, 2-weekly? what should be the form of GSH; face-to-face, remotely? Each participant should, independently, check ONE option for each of the choices. They should hand these in to the facilitators who should tally up the responses and enter them on to a flip chart.

3. Discussion (20 minutes): What are the five most important things that they would do differently from what the research team has suggested?
   - It is suggested that the group uses a brainstorming approach with the chair facilitating the exercise and the facilitator writing the suggestions on to the flip chart.
   - The chair should then facilitate a discussion of the issues that have been raised. Some grouping, splitting, deletions, additions or modification of the issues may occur during this process.
   - The chair should then ask each group member to write the **three most important issues that they would change**. They should then allocate 3 points to the issue that they think is most important to change, 2 points to the second most important, and one point to the change they think is the third most important.
   - Once everyone is ready, the chair should get each group participant to read out their votes and the facilitator should write these up on the flip chart paper.
   - The facilitator or chair should then tally up the scores and announce the five top-ranked changes to the group.
   - The facilitator should write each of these five things on to one of five separate VIPP cards, labelling them 1 to 5 starting from the top-ranked change. The group should hand these to the facilitators who should group them on the wall ready for discussion (see below). One of the facilitators should be doing this while the lead facilitator starts the discussion of the results from the first two tasks.

Section 4: Plenary discussion of proposals for the way forward (20 minutes)
Task 1: Back in plenary, the lead facilitator should summarize the results of the first task (Whether the idea of PREPWELL be taken forward). A brief discussion should follow, especially if there is any disagreement between the three groups.

Task 2: Still in plenary, a facilitator should reveal and summarize the results of the discrete choices and open this for a brief discussion.

Task 3: A facilitator should then summarize the suggested changes using the groupings of related suggestions. A brief discussion should follow, especially if there is any disagreement between the three groups.

Section 5. Wrap-up (10 minutes)
The site PI should then summarize the main outcomes from the workshop.

Thank the participants, facilitators and administrative staff who helped organize the workshop, and wish the participants **a** safe journey home.

5. After the workshop

- Either during the workshop or as soon as possible after the workshop ends, photographs should be taken of the cards and flip charts. These and notes taken during the workshop should be converted into a written summary of all the important outcomes of the workshop including the key points made on the VIPP cards by each group.
- The facilitators should use this as the basis for a written report of the workshop.
