# Supplementary material for: Programme for the Effective Promotion of Maternal Psychosocial Wellbeing (PREPWELL) in Ghana: Development and field-testing of a mHealth Intervention in a rural setting
Source: PLOS Ment Health. 2026 May 19;3(5):e0000571. doi: 10.1371/journal.pmen.0000571 (PMC13186332; doi:10.1371/journal.pmen.0000571)
Supplement: Attachment — Submitted filename: Response to reviewers_PMEN-D-25-00438.docx [file pmen.0000571.s003.docx]

Dear Dr. Li,

PMEN-D-25-00438: Programme for the Effective Promotion of Maternal Psychosocial Wellbeing (PREPWELL) in Ghana: Development and Field-testing of a mHealth Intervention in a Rural Setting.

Thank you for your comments regarding our manuscript and the invitation to revise and resubmit. Please see below a table summarising the response to the concerns raised by the reviewers. All the revisions in the revised manuscript are in track changes or highlighted.

| **Comment/concern** | **Response/action** |
| --- | --- |
| Reviewer 1: To be very honest just needs minor amendments from above suggestions to be very fair, otherwise good to get published. | We thank you sincerely for your encouraging remark |
| 1. The abstract and introduction sounds very robust, clear with strong aims and evidence on arguments on how the approaches to publications, research ethics and publication ethics. 2. I like the use of bar charts and graphs to analyse the mental health conditions of importance to participants in figures 2, 3, 4. Makes more visually appealing to readers and easier information to digest and more engaging to analyse results. 3. Table 2: Evolving Obaatanpa Intervention Framework: Application of core HAP strategies to promote psychosocial wellbeing. Good evidence of research to promote psychosocial well being. 4. I like the structure of the paper and the bold titles and headings to separate the paragraphs and provide concrete substantial evidence of research and data analysis of maternal mental health services for mothers for example, good thorough information and I like the statistics and tables shown to represent the numbers who utilise mental health services | Thank you for these positive observations |
| 5. You could perhaps include some personal case studies or anecdotes from participants who suffered from specific mental health conditions or mothers who have used maternal health services to flesh out the Paper and make it more engaging and interesting to read and adds depth to the analysis of research. 6. You can try to see if you can interview some participants from the study within recent years of service from 2020-2025 to identify who has suffered these conditions and used those services to improve pshycho social wellbeing | Thank you for this important suggestion, which we believe will be useful in the pilot phase of the research programme. As this phase of the research programme was focussed on developing the prototype for *Obaatanpa,* we were limited in collecting follow-up data to evaluate the impact of *Obaatanpa* on the psychosocial wellbeing of the mothers. |
| 7. I would double check spelling, grammar and sentence structure, make sure all citations and references are dated correctly, in alphabetical order according to your style guide and make sentences clear and to point, try to avoid paraphrasing and jargon, adding in useful quotes and include relevant (present day) facts/stastics with your arguments validates your points more especially in the middle of your paper. | Thank you for these useful suggestions. We have now conducted a thorough check for spelling/grammar using available software. We have also improved the report with additional data to support our findings. |
| 8. Also expand on your conclusion, The development process is continuing into phase two of the PREPWELL programme which will involve a large pilot study to further refine Obaatanpa and evaluate implementation outcomes. E.g. Where do you see the improvements of the programme in Mental Health, in five years time in the future in terms of Ghana's social and mental well being, | This is an important question, and one we take seriously. This is particularly important for mHealth interventions that are heavily reliant on advances in technology. We have this in mind and have designed *Obaatanpa* to remain relevant into the future with the flexibility of updating the content of the intervention. We developed *Obaatanpa* to be compatible with the lowest range of mobile phone functionality – a feature phone! This ensures equity and inclusivity for mothers from low SES who may only be able to afford such phones. Beyond this, we are collaborating with the Ghana Health Service to ensure the potential uptake of *Obaatanpa* into Ghana’s comprehensive Reproductive, Maternal, Newborn, Child and Adolescent Health and Nutrition programme – similar to *The Mobile Technology for Community Health in Ghana (MOTECH)* |
| Reviewer 2: |  |
| 1. Style and Format: Kindly ensure the manuscript is submitted as per the suggestions by PLOS Mental Health and the link, particularly:- 2. 1.1.Reference style: change to PLOS uses “Vancouver” style, as outlined 3. 1.2.Abbreviations: some of the abbreviations seem to be missing in the first appearance in the text, refraining from using non-standard abbreviations unless they appear more than three times in the text and keep it minimum 4. 1.3.Result reporting style: Kindly adhere to the format 5. 1.4.Reference list: Please update based on guidelines | Thank you for these helpful observations. We have updated the reference style, results reporting style, and addressed the use of abbreviations to be consistent with standard writing guidelines. |
| 6. 2.1.Page 3, Line 50 -53: Rephrase to academic writing style, e.g. Prevalence of perinatal depression in LMICs is xx% antenatally and xx% postnatally, respectively. | Thank you for the suggestion. What we sought to communicate here is the disparities in the burden of perinatal depression between HIC and LMICs – not necessarily within the perinatal period. We have now revised the sentence to make it clearer. |
| 7. Page 3, Line 59: Please specify the type of treatment | The data points to the lack of any form of treatment (psychotherapy, pharmacotherapy or both). We have clarified to let readers know we’re referring to treatments for mental health conditions which we thought was implied from the preceding sentence. |
| 8. Page 4, Line 81-84: Please rephrase, having limitations to understand. | Thank you. We have now improved the clarity of the sentence. |
| 9. Page 5, Line 96-97: Please follow the reference and citation guidelines | Thank you. Our reference to the chapter and page number is for emphasis and clarity. The actual reference for the source document was provided. |
| 10. Please include the brief demographic characteristics and the issues of Ghana, such as low gadget ownership and data subscription, infrastructure challenges, attrition rate in treatment, and any existing and former digital MH studies conducted in Ghana | Thank you for this observation. We have now included background information specific to Ghana regarding use of mHealth and the evidence gap. |
| 11. Please relook into the Introduction and the sequence. Reading the introduction, readers may have limitations in understanding how this app converges or diverges with existing digital MH platforms or the research gap. | Thank you for this observation. We have now included background information specific to Ghana regarding use of mHealth and the evidence gap |
| 12. Page 6, Line 138, Please provide keywords of literature reviews | This has now been provided |
| 13. Page 8, Line 167-190: Please provide references | We have now provided references to support the use of HAP in Nepal and Uganda. HAP was originally developed in India and this has been appropriately cited. |
| 14. Page 8, Line 184: Please rephrase. Could you help the reader to understand what “ranking (12) and visualisation…” means? | This has been clarified |
| 15. Page 8, Line 187-199: Please summarise, consider including the questions in supplementary material. | Thank you for this useful suggestion. We have summarized the activities in the workshop and will maintain the questions as supplementary material. |
| 16. Page 8. 202-204 Please rephrase. Could you help the reader understand what DA and BW are? Not a common abbreviation. | Thanks. These are codes for the clinical psychologist and the PI who played various roles in the study. We thought it is useful to show this in the manuscript but we have now removed them. |
| 17. Page 10, Line 213-221: Please specify and explain the translation process, if the research employed any linguistic experts or back-to-back translation, etc. | Thank you for this useful suggestion. The study engaged the services of an experienced tech partner to develop the digital platform. The translation process was handled by the tech partner using experienced bi-lingual experts. The research team reviewed the recorded translations for accuracy and completeness before these were programmed for deployment.  We have clarified the process a bit more in the manuscript. |
| 18. Page 11: Please specify the participants' recruitment inclusion and exclusion criteria and how the PHQ was administered and why the PHQ was administered, as well as the analysis. PHQ has a cut-off score and provides a brief introduction of PHQ under the instrument sub-section. Are your studies looking for patients with depressive symptoms? | As this is an exploratory formative study, we considered only minimal inclusion/exclusion criteria. These were provided (line 253): ‘Aside from pregnancy and postnatal status, other eligibility criteria included: access to a mobile phone and being able to understand the Twi language’  As explained in the manuscript the PHQ-9 was administered for the sole purpose of assessing the safety (no severe symptom deterioration) of the *Obaatanpa* prototype. Participants were not recruited/included in the field-testing based on their PHQ-9 scores (*Obataanpa* is designed for universal prevention), thus we did not have to use cut-off norms even though these exist in the study site. |
| 19. Page 12: Please provide a sample size calculation or power analysis. | Thank you for this comment. We understand that sample size and power calculations are essential for hypothesis-testing trials; however, this study is exploratory and formative, focused on developing and refining a prototype intervention rather than testing efficacy. As such, we did not conduct a formal power calculation. Instead, our sample size was determined pragmatically to ensure sufficient diversity and depth for qualitative exploration and iterative co-design, consistent with guidance for formative and usability-focused studies. |
| 20. Please re-examine the flowchart, suggesting drafting a flowchart with CONSORT | Thank you for this comment. We understand the importance of flowcharts/CONSORT diagrams as they are an effective summary of study conduct; however, in this small but complex exploratory/formative study, we focused on showing how the multiple steps were executed for the purposes of replication. |
| 21. Page 14, Line 286-299: Please focus on result reporting | Thank you for this comment. In the section referred to, we reported the findings from our review of key contextual information to inform the design of *Obaatanpa* |
| 22. Page 15-17: Please rephrase and follow the result reporting guideline. | Thank you for this comment. In the section referred to, we reported the findings from our review of key contextual information to inform the design of *Obaatanpa.* For example, we shared synthesized findings on acceptability of mobile phone delivered mental health promotion intervention from our formative workshops with participants. Participants were clear that the messages should be comprehensible ('easy to understand') etc  We also presented findings using charts on: preferred mental health conditions, preferred HAP strategies, and psychosocial domains of importance to participants. |
| 23. Page 18/ Table 2: Please check the reporting guideline and relook into the title of the table and table formatting. Please provide the phrase and the number of sessions for each segment in your study. | Thank you for your comment.  Table 2 is designed to describe the theoretical framework of the *Obaatanpa* intervention prototype. It shows how *Obaatanpa* maps on to HAP strategies and psychosocial wellbeing domains.  We have slightly edited the title to capture the essence of a framework. |
| 24. Please check the page number and the line number; all are missing. Please rephrase and follow the result reporting guideline. | Thank you for alerting us to the line numbering issue. It’s not immediately clear to us what explains this but we have tried to fix it. |
| 25. The PHQ result was mentioned without a table, and the comparison result. Is PHQ scoring part of the measurement of the effectiveness of the study? | As explained above and in the manuscript the PHQ-9 was administered for the sole purpose of assessing the safety (no severe symptom deterioration) of the *Obaatanpa* prototype. This is not an effectiveness study. |
| 26. Please relook into the mental health digital app development stage/ study design, the intervention framework shall follow a structured & clear study design that spans several phases. Some of the phrases are achieving different goals, and the development process is unclear. Upon reviewing your manuscript, I suggest breaking it into 5-7 Phrases. - Phase (1) Need assessment with mixed method, e.g. Stage A … Stage B…; Phase (2) Baseline; Phase (3) Programme development; Phase (4)…; Phase (5) Implementation of trials; Phase (6) Follow-up and evaluation. | Thank you for the invitation to make the report clearer regarding the process of *Obaatanpa.* We appreciate that this falls within the realm of complex interventions and we tried to follow the guidance from the Medical Research Council Framework for developing and evaluating complex interventions.  We did this by laying out three main stages and multiple steps of research (a) formative research to identify key contextual information regarding the feasibility and acceptability of the *Obaatanpa* intervention; (b) constructing a prototype *Obaatanpa* by adapting an evidence-based psychological treatment known as the Healthy Activity Programme (HAP) to identify specific components that are feasible and acceptable to mothers and other stakeholders; and (c) field testing the prototype intervention. We included a figure (fig 1) to summarize the stages and systematic steps in developing and field-testing the *Obaatanpa* prototype. |
| 27. Please consider adapting the CONSORT-style flowchart to visualise the study design, including the number of sessions and outcome/ goals. | Thank you for your suggestion. We agree this is a complex study and it’s important to make things clearer for readers.  We did indeed adapt a ‘CONSORT-style’ flowchart to show the study flow. We presented this information in a figure – figure 1: **Overview of the development process of *Obaatanpa*** |
| 28. Please consider providing a table for Fig. 2-4 with frequency and descriptive data, instead of a graph. Fig. 5 is missing, or the file is unreadable. | Thank you for this suggestion. Given the nature and quantum of data, we felt using visuals to tell the story might be more effective in communicating the data.  We thank you for letting us know about fig 5 – we now realize the file is corrupted and have provided a new one. |
| 29. Please help the readers understand why gadget ownership was discussed, but the result section was not highlighted | Thank you for this comment. We identified the importance of phone ownership in stage 1: Identifying key contextual information on maternal mental health services; step A: identification of digital interventions for mothers. In the results informed by the evidence synthesis, we wrote this ‘A synthesis of these studies suggests important learnings for the uptake of technology enabled public health interventions: a) it is important to consider mobile phone ownership dynamics….’  This provided the context for our reference to phone ownership in our discussion. |
| 30. Please help the readers understand why help-seeking behaviours or self-care behaviours changed after the intervention, and based on your findings. | Thank you for this comment. We did not seek to evaluate these important outcomes at this stage of the development process – these will be addressed in the next phases of the PREPWELL programme of research when we will employ appropriate experimental designs.  Our outcomes aligned to field-testing of the prototype *Obaatanpa* intervention included: Acceptability, Feasibility, Comprehensibility, Saliency, and Safety of *Obaatanpa* |
| 31. Please provide references to support your findings and split them into a few paragraphs based on your findings, e.g., the clean interface of the apps and the narrative-based intervention. | Thank you. We believe this comment is directed at our discussion.  Given the data in this report is exploratory/formative work on developing a digital technology-supported psychological intervention, our discussion focussed on highlighting key processes and learnings, including the theoretical basis of our intervention.  We first reflected on the growing value of mHealth interventions and how our PREPWELL programme through *Obaatanpa* is adding to this literature base – appropriately referenced.  We discussed the theoretical stance from the lens of positive psychology and how we can leverage interventions originally designed from a treatment framing (HAP) for use as preventative/promotive interventions – supporting WHO’s self-care approach to health promotion – appropriately referenced. We discussed the approaches used in *Obaatanpa* to optimise acceptability e.g. use of storytelling – appropriately referenced. We also discussed the core elements of *Obaatanpa* such as use of problem-solving and the supporting evidence that we cited. We discussed the appropriateness of HAP for *Obaatanpa* and the probable mechanisms explaining its role in promoting well-being – drawing and referencing Seligman’s proposed components of a happy life.  We also discussed pragmatic issues related to phone ownership and the opportunities the increasing penetration of mobile phone ownership in LMIC presents for interventions of this nature. Tied to this, we discussed our learning about how to deliver such interventions to make them more acceptable for a largely illiterate population – we acknowledged these challenges are not new in LMIC settings – this was appropriately cited. |
| 32. Please explain the effectiveness of the apps, for example, whether the content helped the participants who had low media literacy easily grasp the self-help tips. Please provide a reference. Please help the readers understand what “promising result” is. | Our characterization of the results from this field-testing stage as ‘promising’ is grounded on the findings on our main outcomes – Acceptability, Feasibility, Comprehensibility, Saliency, and Safety.  Overall, the evidence points to *Obaatanpa* as potentially acceptable to mothers, feasible to implement and receive, comprehensible in terms of the messages and activities, meaningful to mothers, and more importantly safe. We represented this in the discussion with this text: **‘Our findings show the initial promise of a feasible and acceptable technology-delivered self-help intervention that seeks to empower mothers to apply simple, evidence-based strategies to help improve and maintain their mental health during periods of heightened vulnerability’**  As clarified in a similar comment above, effectiveness (clinical/cost) was not an outcome in this stage of developing the prototype. For this reason, we did not discuss effectiveness. However, the qualitative accounts from the mothers indicated that in a low literacy population as ours, the mothers were able to understand the instructions on how to interact with *Obaatanpa* on their phones. We reasoned that this was possible because we used the interactive voice response option – the mothers were clear they could not read SMS type messages. We also used storytelling – and we provided a reference to this. The qualitative accounts also demonstrated that mothers were able to understand and use some tips. For example from the data: “*Obaatanpa said when we are worried over issues, we should try and talk to friends.*  *It helped me to stop some fears and thinking associated with pregnancy and childbirth”* |
| 33. Please explain how the apps link with positive psychology? If positive psychology is part of the theoretical framework, please include it in the study design. | Thank you for this important comment.  The theoretical foundation for *Obaatanpa* is Behavioural Activation (BA) Therapy. This is the underpinning theory behind the Healthy Activity Program (HAP) that we adapted for *Obaatanpa.* We note elements of positive psychology in BA theory and thus provide the grounding for our reasoning that HAP (originally designed for targeted treatment of depression) can be adapted for promotion of psychosocial wellbeing.  The introduction articulated these points and we have further clarified the HAP intervention and its BA stance in the methods section. |
| Reviewer 3 |  |
| The manuscript demonstrates strong methodological and ethical rigor, offering a valuable contribution to global mental health research. Minor revisions are advised to improve language consistency and overall clarity. | Thank you for your positive feedback |
| Reviewer 4 |  |
| The abbreviation BA is not written in its full form. Please write the full form of BA intervention in the introduction to clearly state its purpose. Also a little detail can be added in the introduction section about BA interventions explaining what they are. | Thank for this comment. We have now provided a short description of BA. |
| In the introduction section the BA intervention is linked to promoting psychological wellbeing but the population is not specified. Is it the pregnant women of the general population that BA interventions had shown improvements with. Perhaps the idea of BA interventions linking to maternal psychological wellbeing should be made clear. | We have now clarified the population in Mazzucchelli meta-analysis – this included studies from the general population and not specifically on pregnant/recently delivered mothers. The literature has not shown evidence of BA used specifically promoting maternal psychosocial wellbeing – there’s evidence of BA as treatment for perinatal depression – a component of psychosocial wellbeing.  This is the gap our study seeks to address. |
| The introduction section does not state what mental health problems are present among women who are pregnant. And also, the current intervention is designed for which mental health problems. (eg depression, anxiety, major depressive disorders etc.) | In our definition of maternal psychosocial wellbeing in the introduction (paragraph 1), we clarified that this includes perinatal depression at one of its extremes.  The intervention we’ve developed is designed to promote maternal psychosocial wellbeing – and this includes depression. In the next phase of the PREPWELL program we will design appropriate measures of psychosocial wellbeing and this would include: distress, anxiety, depression, coping, problem solving, and social outcomes (e.g., family and community support, empowerment, culture) |
| Clearly state if the intervention is a type of BA intervention or something else. (clearly mention that BA is a type of evidence-based psychological treatment which this intervention will follow) | We have now clarified this in both in the introduction and methods. |
| Write the full form of HAP in the introduction section where it is first introduced | We have provided the name in full |
| Clear rationale for why this evidence-based treatment was chosen | We provided this information in the methods section under stage 2 of the development process |
| Use abbreviations correctly throughout the manuscript | We have now streamlined the use of abbreviations |
| Inclusion/exclusion criteria is not clearly defined (target population, age group, mental health condition, language, target sample size etc.) | At each stage and step of developing the prototype intervention, we specified what data were collected and from who/what.  For example, in our rapid review to identify digital health interventions for mothers, we provided information pertaining to the search strategy.  For our workshops with various stakeholders, we identified who participants were and how they were identified. For example we provided this information: ‘workshops were conducted in three communities purposively selected for ease of access and presence of a community health nurse’  For our field-testing to evaluate the prototype intervention we provided information on who participants were and how they were identified. For example, we wrote this: ‘small group (8 pregnant; 8 recently delivered mothers) of mothers attending antenatal and postnatal care clinics in one health facility in the study area. Aside from pregnancy and postnatal status, other eligibility criteria included: access to a mobile phone and being able to understand the Twi language’ |
| Recruitment method not described (community, clinics, etc.) | We provided this information: ‘small group (8 pregnant; 8 recently delivered mothers) of mothers **attending antenatal and postnatal care clinics** in one health facility in the study area’  Just to clarify again that as this is an exploratory/formative study to develop an intervention, we were more purposeful and pragmatic in our approach to identifying participants. |
| )The manuscript mentions that a rapid review was conducted to gather existing evidence on the topic; however, the review itself is not cited, published, or made accessible. For transparency and reproducibility, please clarify: 1) Whether the review followed a protocol (and if so, provide registration details or an appendix). 2) Whether the review results are available (e.g., as supplementary material, institutional report, or online dataset). 3) The databases searched, inclusion/exclusion criteria, and search terms. | The rapid review was conducted as step A (**identification of digital health interventions for mothers**) of stage 1 (**Identifying key contextual information on maternal mental health services)** of the development process. As explained in the manuscript, we opted for the rapid review approach to narrow the scope of the research question and reduce the extent of data abstraction to provide evidence in a timely and cost-effective manner particularly because we had limited resources to conduct a full systematic review. To this end, we did not feel it was necessary/required to have this registered as is usually required for other forms of evidence synthesis such as scoping and systematic reviews.  We provided details of the search strategy and the databases used. |
| The introduction for HAP should be moved to introduction section and made clear with the objectives of the study. | Thank you for this suggestion. HAP is founded on Behavioural Activation theory and we provided the relevant literature to support this. We reserved the detailed description of HAP for the methods section as we felt this is where it should be – description of the intervention for the planned adaptation.  We stated the objectives of the study in the objectives as: The development process as described was guided by the following objectives: 1) to adapt a BA-based psychological treatment – Healthy Activity Program (HAP) into a culturally acceptable guided self-help psychosocial well-being intervention for delivery to pregnant women and mothers who have recently given birth in the Kintampo North Municipality of Ghana; 2) to assess *Obaatanpa*’s feasibility, comprehensibility, acceptability, and saliency for pregnant women and women who have recently delivered in the Kintampo North Municipality of Ghana. |
| There is no step C? | Thank you for letting us know the labelling of step C is missing in the description of the process. There is a step C and we mistakenly skipped that for step D. We have now corrected the sequence to include step C which was part of stage 2 of the development process. Stage 1 had two steps – A and B. |
| )Mention the steps in the stages in the paragraph. Eg, the first stage involves 3 steps: (A), (B), (C) and then start describing the steps to make things clear. | We have now provided clarity regarding the specific steps in each stage to help readers follow the process. |
| Use proper and similar referencing in the manuscript | We have now streamlined the referencing in the manuscript. |
| The manuscript mentions the use of “structured workshop guides” to conduct participatory learning and action sessions. However, the source and development process of these guides are not described. For transparency and replicability, please specify: Whether the guides were adapted from existing manuals, developed, or co-created with participants. How the content was structured (number of sessions, key topics, activities). Whether the materials are accessible (e.g., in supplementary materials or upon request). | Thank you for this comment.  We have now provided some detail to clarify how the guides were developed. In terms of how these were used during the workshops and the topics discussed, we provided the following information in the manuscript: ‘For example, structured workshop guides were used to guide the completion of four key activities: indicating yes or no regarding previous experience of seeking help from a health worker for issues that impact on mental health (e.g. Have you ever been to a health worker to discuss something that is bothering you and to get help in terms of counselling on how to deal with the problem during **pregnancy or when you have given birth**?) ; indicating yes or no regarding willingness to receive counselling (e.g. Would you like the opportunity to receive help on **how to take care of your own mental health** during pregnancy and after delivery or for a mental health problem in general?; ranking and free listing for preferred content (e.g. What mental health conditions/areas would you like this programme to focus on?; What psychosocial well-being areas would you like PREPWELL to focus on?; What elements of the Healthy Activity Programme would you like PREPWELL to focus on?); (b) consensus-building workshop with experts and practitioners in maternal and mental health, mental health user groups, and Ghana Health Service stakeholders. |
| The manuscript describes the use of participatory approaches (e.g., PLA techniques), but there is no mention of patient or public involvement (PPI) in the design, conduct, or interpretation of the study. Since participatory research inherently values collaboration with community members, it would strengthen the manuscript to clarify: Whether and how target participants or community representatives were involved in shaping the study design or materials, and If not, to include a formal statement on the absence of PPI in accordance with journal guidelines. | This is an important comment and one we take seriously.  *Obaatanpa* was co-created with key stakeholders including end-users such as the mothers and service providers, including health service administrators – we made an explicit mention of this in the introduction to stage 2. Our systematic approach to developing *Obaatanpa* was guided by this principle from the outset. In stage 1, step B we consulted health directors, public health nurses, midwives, community psychiatric nurses, clinical psychologists, community mental health officers, community psychiatric officers, community health planning services coordinators, disease control officers through key-informant interviews. This information shaped our engagements with the mothers in the workshops. The workshops with mothers were designed to be participatory by providing mothers the power to decide what they wanted *Obaatanpa* to do for them. They told us what mental health areas we should focus on addressing and what psychosocial wellbeing domains should be included in designing the intervention. As outlined in step D, we held a consensus-building workshop with representatives of the end-users, and health service administrators to validate the evolving content and mode of delivery of the intervention. |
| Citation for the original PHQ-9 paper (Kroenke et al., 2001)? Did you use PHQ-9 as an outcome measure (to assess mental health before and after the intervention)? Was it self-completed or interviewer-administered? How were the incomplete responses handled? | As explained in the manuscript the PHQ-9 was administered for the sole purpose of assessing the safety (no severe symptom deterioration) of the *Obaatanpa* prototype – one of five outcomes we assessed in the field-testing – Acceptability, Feasibility, Comprehensibility, Saliency, and Safety  It was interviewer-administered, and we did make this clear in the manuscript – ‘Assessments using the locally validated Twi version of the Patient Health Questionnaire-(PHQ-9) [24] before and after receiving *Obaatanpa* were **interviewer-administered** to check safety (no severe symptom deterioration)’.  There were no incomplete responses. |
| The manuscript reports on an intervention but does not specify the study design (e.g., randomized controlled trial, quasi-experimental, pre–post, or pilot). Clearly identifying the research design is essential to understand the level of evidence, sampling method, and appropriate analysis. Please clarify the study design and describe any comparison or control groups, randomization procedures, or rationale for the chosen design | Thank you for this comment. We understand that providing information on design considerations and processes are essential for hypothesis-testing trials; however, this study is exploratory and formative, focused on developing and refining a prototype intervention rather than testing efficacy. As such, we did not conduct a trial or an experiment. Instead, our design is exploratory/formative studies for intervention development and we did follow the Medical Research Council Framework for developing and evaluating complex interventions. |
| The manuscript does not describe the recruitment process, including how participants were identified, approached, and enrolled, nor does it report how many were eligible versus how many participated. Providing this information, including inclusion/exclusion criteria, recruitment methods, and a flow diagram, is essential for transparency, assessment of potential selection bias, and reproducibility. (a CONSORT-style flow diagram) | Thank you for this comment. We understand that providing information regarding these elements/aspects are essential for hypothesis-testing epidemiological studies; however, as mentioned above this study is exploratory and formative, focused on developing and refining a prototype intervention rather than testing efficacy or internal/external validity, for which these aspects of study conduct would have been critical. Instead, our design is exploratory/formative studies for intervention development and we did follow the Medical Research Council Framework for developing and evaluating complex interventions.  Having clarified the study design, we did provide information on inclusion/selection criteria for aspects of the formative study. For example, for the rapid review, we provided information on included studies and our search strategy. We have now included the period of the review based on your comment. For our co-creation participatory workshops, we provided a rationale for selecting the communities that were involved – ‘workshops were conducted in three communities purposively selected for ease of access and presence of a community health nurse’. In the field-testing, we identified the criteria for participation – ‘a small group (8 pregnant; 8 recently delivered mothers) of mothers attending antenatal and postnatal care clinics in one health facility in the study area. Aside from pregnancy and postnatal status, other eligibility criteria included: access to a mobile phone and being able to understand the Twi language’ |
| The information regarding the intervention was not sufficient (time and duration, place, if participants were compensated etc) | We provided a detailed description of the intervention and how it was deployed in step E – developing the digital platform’  We have now expanded on what went into step F in terms of what the mothers were told regarding what to expect and the duration of each session.  Mothers were not compensated for participating and this was communicated in the consent form. Mothers were however told that they will not get charged for responding to the automated calls and interacting with the *Obaatanpa* mobile phone-based intervention. |
| The Methods section requires significant improvement for clarity and structure. Currently, the sequence of information is not well aligned — details about the intervention, data collection, and analysis appear intermingled, and the overall flow does not follow a logical order. I recommend restructuring the Methods section into clear subsections (e.g., Study Design, Setting and Participants, Intervention, Data Collection, Data Analysis, and Ethical Considerations). Each step should be described sequentially and in enough detail to allow replication | Thank you for this comment. We understand that presenting information in the way you have suggested is essential for a typical epidemiological study; however, as mentioned above this study is exploratory and formative, focused on developing and refining a prototype intervention. We presented/structured the methods to reflect the step-wise iterative process as is typical with exploratory/formative studies (that employ multiple methods) for intervention development and aligned with the Medical Research Council Framework for developing and evaluating complex interventions. |
| The study reports that 59 participants were enrolled, but no explanation is provided regarding how this number was determined. Please clarify whether this was based on a formal sample size calculation, recruitment feasibility, or convenience sampling. Additionally, provide context regarding eligibility, recruitment, and attrition to help readers interpret the adequacy and representativeness of the sample | Thank you for this comment. We understand that sample size and power calculations are essential for hypothesis-testing studies; however, this study is exploratory and formative, focused on developing and refining a prototype intervention rather than testing efficacy. As such, we did not conduct a formal power calculation. Instead, our sample size was determined pragmatically to ensure sufficient diversity and depth for qualitative exploration and iterative co-design, consistent with guidance for formative and usability-focused studies. |
| The stage 1 says ‘The technology was evaluated to assess the platform’s effectiveness in delivering messages along with user response across sites in five districts from 2011 to 2014.’ But the study time period was mentioned between 2nd September 2022 and 27th October 2022. Which time period is the correct one? | We are sorry if the dates sounded confusing.  What you are referring to is part of the findings from stage 1 – the rapid review. This was in specific reference to the MOTECH intervention that was deployed in Ghana and its evaluation between 2011 and 2014.  To clarify, our study that we are reporting took place between 2nd September 2022 and 27th October 2022. |
| If the intervention was to provide mental health services for mothers why were mental health assessment mot conducted? | Thank you for this important comment.  The intervention *Obaatanpa* is designed to promote psychosocial wellbeing of women during pregnancy and after delivery. As explained in the background, the dimensions of psychosocial wellbeing include mental health, this case depression.  *Obaatanpa’s* strategy in achieving this is to empower mothers to apply simple, evidence-based strategies – such as HAP to help improve and maintain their mental health during periods of heightened vulnerability.  In terms of evaluation, as explained, this phase of the PREPWELL programme is the development of the prototype intervention, and the interest is not to assess effectiveness outcomes. We assessed mental health – depression only for the purposes of assessing the safety of the intervention. |
| What statistical analysis method was used for result analysis? | For the structured participatory workshops, most of the data were quantitative in nature. These were tallied and ranked. Furthermore, the intervention usage data were collected through a built-in survey on the digital platform. All quantitative data were analysed using simple descriptive statistics to summarize information using counts, frequencies, means and proportions. |
| The data from the analysis is not reported sufficiently. | Thank you for this comment. We understand that a detailed analysis is essential for scientific reporting; however, this study is exploratory and formative, focused on developing and refining a prototype intervention. As such, most of the data – a mix of qualitative and quantitative were quite basic and less complicated. The qualitative data from the field-testing of the intervention were subjected to rigorous qualitative analysis using the framework analysis approach. We described this in the data analysis section. The quantitative data from the workshops based on binary options were subjected to tallies and ranking. The demographic characteristics of mothers in the field-testing study were reported as frequencies and proportions. The PHQ-9 results were also analysed and reported as a mean score across the 8 sessions. A similar approach was used to estimate the mean duration of sessions. |
| Information regarding the data analysis and the method used should be clearly mentioned in the method section under statistical analysis section which was missing | Thank you for your suggestion. We have clarified this concern above. |
| The number of participants is not consistent throughout the result section and it is confusing to interpret how many participants were involved in the intervention and how many dropped out. (in the starting it mention there are 8 pregnant women and 8 recently delivered but in the later sections it mentions ‘8 out of 13 mothers ‘.) | We apologise if the number of participants in the intervention and session completion is confusing.  As described the intervention started with 16 mothers (8 pregnant women and 8 recently delivered).  As shown in figure 5, one participant was deemed to have dropped out after failing to respond over two consecutive sessions (drop-out). Subsequent calls from session 3 were directed at the remaining 15 mothers. By session 5, two more drop-outs were recorded and the calls were directed at the remaining 13 mothers. The 13 mothers remained active until session 8 when 2 drop-out.  The findings we shared took into consideration the total number of participants at that point. For example, the finding that ‘Whilst the majority (11/15) of the mothers knew how to use phones, some experienced challenges interacting with *Obaatanpa,* particularly in navigating between sessions by pressing buttons on the phone’ was based on data collected on the ‘app’ at session 1 (one mother did not respond). Similarly, the finding that ‘8 out of 13 mothers were able to identify that what they do affects how they feel, illustrating some understanding of the connection between actions and feelings’, was a response to an activity completed at session 3. And by session 3 there were 13 active mothers. |
| The Discussion section mentions that the study co-created a prototype psychosocial wellbeing promotion mHealth intervention. However, the Methods section does not describe how this co-creation process was conducted — including who participated, the steps taken, or how findings informed the prototype. As co-creation is a key outcome and methodological process, it should be explicitly described in the Methods, with sufficient detail to ensure transparency and reproducibility. | This is an important comment and one we take seriously.  *Obaatanpa* was co-created with key stakeholders including end-users such as the mothers and service providers, including health service administrators – we made an explicit mention of this in the introduction to stage 2. Our systematic approach to developing *Obaatanpa* was guided by this principle from the outset. In stage 1, step B we consulted health directors, public health nurses, midwives, community psychiatric nurses, clinical psychologists, community mental health officers, community psychiatric officers, community health planning services coordinators, disease control officers through key-informant interviews. This information shaped our engagements with the mothers in the workshops. The workshops with mothers were designed to be participatory by providing mothers the power to decide what they wanted *Obaatanpa* to do for them. They told us what mental health areas we should focus on addressing and what psychosocial wellbeing domains should be included in designing the intervention. As outlined in step D, we held a consensus-building workshop with representatives of the end-users, and health service administrators to validate the evolving content and mode of delivery of the intervention. |
| The section had some point not mentioned before (eg story telling technique was employed). This should be included in the method and result section with data mentioned from the participants. | Thank you for this comment.  We did identify storytelling as a culturally appropriate technique that could enhance acceptability of the intervention at the **design stage**. For example the triangulated findings from stage 2, on the emerging *Obaatanpa* intervention framework identified storytelling as an important feature of the intervention – ‘The main delivery feature for the content is IVR, including the use of story-telling’ – this is also mentioned in table 2; and in **the results**. For example, under acceptability of the intervention, we stated the following: ‘It appears the use of the story-telling approach in delivering the intervention was an important ingredient for the acceptability of *Obaatanpa.’* |
| The data was only given in graphical form and should also be given in the numerical form for transparency | Thank you for this suggestion. Given the nature (multiple counts on a given variable) and the small sample, we felt using visuals to tell the story might be more effective in communicating the data. We ensured to include the data points in the charts. |
| Final Evaluation despite the relevance and potential impact of the study, the manuscript suffers from major methodological and reporting shortcomings that compromise its transparency, reproducibility, and scientific rigor. The paper requires significant revision and clarification before it can be considered for publication. Clearly identify the study design and align the methodology, data collection, and analysis accordingly. Expand the Methods to include recruitment, eligibility criteria, sample size rationale, and detailed procedural steps. A CONSORT-style flow diagram would strengthen reporting transparency. Describe the analytical process in sufficient detail, including data preparation, coding, thematic development, statistical tests (if applicable), and software used. Include a section describing how participants or community members were involved in co-design or decision-making processes. Provide complete numerical data in tables, including descriptive and inferential statistics. Graphs should complement—not replace—numeric reporting. Move or expand this content into the Methods section and describe the co-creation process systematically. | We thank you for your encouraging comments about the relevance of this work – which we agree completely.  We are encouraged by the thoroughness of your comments and the opportunity for us to respond.  We are hopeful our responses to each of your comments has helped clarify areas of concern but also improved the manuscript. |

I look forward to your reply in due course.

Sincerely,

Benedict Weobong
