# Supplementary material for: Programme for the Effective Promotion of Maternal Psychosocial Wellbeing (PREPWELL) in Ghana: Development and field-testing of a mHealth Intervention in a rural setting
Source: PLOS Ment Health. 2026 May 19;3(5):e0000571. doi: 10.1371/journal.pmen.0000571 (PMC13186332; doi:10.1371/journal.pmen.0000571)
Supplement: Attachment — Submitted filename: Response2 to reviewers_PMEN-D-25-00438.docx [file pmen.0000571.s004.docx]

Dear Dr. Li,

PMEN-D-25-00438: Programme for the Effective Promotion of Maternal Psychosocial Wellbeing (PREPWELL) in Ghana: Development and Field-testing of a mHealth Intervention in a Rural Setting.

Thank you for the additional comments regarding our manuscript and the invitation to revise and resubmit. Please see below a table summarising the response to the concerns raised by the reviewers. All the revisions in the revised manuscript are in track changes or highlighted.

| **Comment/concern** | **Response/action** |
| --- | --- |
| Reviewer 1: |  |
| 1. My only little concerns/last edits to the paper to make it seem outstanding.   is maybe adding some more anecdotes or personal experiences or accounts of women in Ghana who have been through maternity in rural settings facing through psycho social mental health problems and how mental health interventions can help them and monitor/assesses their mental health needs in terms of digital technology guiding them in rural areas too in helping them farming or better improve their life styles, through getting some interviews or case studies of personal video questionnaires just to flesh out the paper a bit more, and make it flourish with some qualitative perspectives and humanising the paper, may invite more readership to connect to the paper too.  i.e. how their personal experiences, how are they coping with those conditions/ specific symptoms, is there enough done to ensure the mental health interventions can help them in Ghana with psychosocial well being and mental well being in helping them attend more skills learning classes, community grass roots maternity focus groups with other mothers who are going through similar experiences, so all the mothers are not left isolated or alone in rural communities, to help attend group social activities to make and meet new friends/like minded people, in rural settings for all mothers too, i.e. supportive of rural businesses together, cooking, art or textiles, sports/yoga/reiki/meditation, music/dance, or fashion or teaching new skills i.e reading books, learning computers/PC etc. | Thank you for these useful suggestions, that we believe can enhance the story we are telling in this study. Your suggestion to add interesting accounts from women is welcome and we will consider this in the next phase of the PREPWELL program. For example, we may consider adding a module to address social determinants of mental health through the power of self-help groups – this model of community mental health is strongly demonstrated in the Friendship Bench program. Given the study has ended, we are constrained about collecting additional data at this stage. However, what we have done is to include more quotes from the mothers regarding their experience of pregnancy-related mental health challenges*.* For example, we included the following text in the results: **‘To buttress this call and willingness for counselling services, the mothers shared a troubling incident where a woman who had recently given birth almost killed her baby because of her poor mental health – in their own words *‘it really troubled and disturbed the family so, she thinks it is important to seek help’*** |
| 2. You can include in conclusion too, perhaps if you want the next future steps on how the Mental Interventions will help progress positivity in mental well being in Ghana.   And forwardness in encouraging Ghana's rural public to help psycho social well-being especially in Maternity to help empower their mind, body spirit by introducing yoga/reiki/meditation to be very fair to keep the positive mental health for future years to come, e.g. in next 5 to 10 years Ghana's maternal program of development is progressed by these mental health interventions and helped specifically the mother's psychosocial well being by these solutions i.e. mentioned grass roots projects, opening up community classes, social activities, more digital technology in medicine advanced to help aid and measure the mental health of mothers in Ghana, using perhaps new inventions or interventions like mental health monitors of maternal patients in pharmacies/ hospitals etc. to assess and measure the mental health needs of mothers more accurately, which in rural areas can be invented and distributed free in those communities, to assess happy/sad/anxiety/mood disorders/social/emotional levels of mental health too for example. | Thank you for this important suggestion. We have no highlighted some of these points in the conclusion. |
| Reviewer 3: |  |
| 3. Claims related to scalability and broader impact in the discussion should be framed more cautiously to align with the small sample size and exploratory nature of the field-testing phase. With minor revisions to strengthen interpretive balance, the manuscript is suitable for publication. | This is an important suggestion. We have now toned down on the language around scalability in the abstract to reflect the exploratory nature of this study. |

I look forward to your reply in due course.

Sincerely,

Benedict Weobong
